# Brachytherapy treatment planning commissioning: effect of the election of proper bibliography and finite size of TG‐43 input data on standard treatments

**DOI:** 10.1120/jacmp.v16i4.4730

**Published:** 2015-07-08

**Authors:** Christian N. Valdés, Gustavo H. Píriz, Enrrique Lozano

**Affiliations:** ^1^ Departamento de Física Médica Centro Oncológico de Antofagasta Antofagasta Chile; ^2^ Departamento de Física Médica Centro de Oncología ONCOSUR Florida Uruguay; ^3^ Departamento de Física Médica Instituto Nacional del Cáncer Santiago Chile

**Keywords:** HDR brachytherapy, quality assurance, commissioning, interpolations, proper references

## Abstract

The aim of this work is to evaluate the performance of a commercial brachytherapy treatment planning system (TPS) with TG‐43 Vendors Input Data (VID), analyze possible discrepancies with respect to a proper reference source and its implications for standard treatments, and judge the effectiveness of certain widespread recommended quality controls to find potential errors related with the interpolations of TG‐43 VID tables. The TPS evaluated was a BrachyVision 8.6 loaded with TG‐43 VID for a VariSource high‐dose‐rate 192Ir source (Vs2000). The reference data chosen were the TG‐43 data published in the literature. In the first step, we compared TG‐43 VID with respect to the chosen reference data. Next, we used percent dose‐rate differences in a point array matrix to compare the outcomes of the TPS on standard treatment setup with respect to an in‐house developed program (MATLAB R2009a‐based) loaded with the chosen full TG‐43 reference data. The cases with major discrepancies were evaluated using the gamma‐index analysis. The comparison with the reference data indicated a lack of sample in the angles between near to the tip (between 165<θ<180) and cable (0<θ<15) of the $F{\left(r,\theta \right)}_{\text{VID}}$, which causes a dose underestimation of approximately 17% in the investigated points due to inaccurate interpolations. The differences over 2% encompassed approximately 17% of the surrounding source volume. These results have special relevance in treatment using one applicator with a few dwell steps or in Fletcher treatments where 10% dose underestimates were identified within the tumor or in organs at risk, respectively. Our results suggest that the differences found in the TPS under study are created by a lack of information on the angles in high‐gradient zones in the $F{\left(r,\theta \right)}_{\text{VID}}$, which generates important differences in dosimetric results. In contrast, the gamma analysis shows very good results (between 90% and 100% of passed points) in the analyzed treatments (one dwell and Fletcher). Further studies are required to exclude the possibility of finding noticeable effects in the DVH of treatment plans caused by the discrepancies here described. To achieve more strict control over the TPS dose‐rate calculation, we recommend using QA test thinking in a source with nonaxial symmetry, adding a control point on the angles of the high‐dose gradient zones (e.g., between 0° and 15° and between 165° and 180°). More studies are required to achieve full understanding of the clinical implication of such discrepancies.

PACS number: 87.55.Qr

## I. INTRODUCTION

Brachytherapy has an important role in gynecological radiation treatments, and there has been a great amount of clinical experience accumulated over the years regarding it. Nevertheless, with the introduction of the high‐dose rate (HDR) remote afterloaders, it has achieved highly widespread practice. Some inherent advantages of this technology are the reduction of the exposure of personnel, optimization of the dose delivered the target and organs at risk, and ambulatory treatments possibilities, among others.^(1)^ However, each advantage brings with it certain drawbacks, such as the requirement for complicated equipment, specialized personnel with longer training, and the increased needs for accurate dosimetry, which requires the use of computerized treatment planning systems (TPS).^(1)^

Although there has been a recent introduction of new algorithms based on Monte Carlo methods, collapsed cone methods, and grid‐based Boltzmann transport equation solvers,^(2)^ there is a broad consensus about the use of the TG‐43^(3)^ formalism as a basis for the dose calculation around a brachytherapy source.^(4)^ One of the major improvements of this formalism is the use of specific sources factors that take into account the source‐to‐source differences in encapsulation and internal construction.^(5)^ Thus, all TPS require entry data with the factors of each source used in the clinic.

It is quite common for TPS to come with vendor input data (VID) obtained from Monte Carlo simulations. Although the major portions of the TPS use the same formalism, each system has small differences in the methods associated with, and the quantity of, the entered data (e.g., numbers of angles in the 2D anisotropy function table, use of polynomial fit or interpolations in the radial dose function). The finite size of the TG‐43 entry data may cause outcome discrepancies due to interpolation (ODIN) between the brachytherapy treatment planning system (BTPS) calculation and the expected value (often obtained from the same reference source as the VID) over the 2% recommended by the AAPM.^5^, ^6^

Despite the simplicity of the TG‐43 formalism, several organizations recommend performing a complete acceptance and commissioning procedure before its clinical use as an essential part of brachytherapy QA programs,^1^, ^4^, ^7^, ^8^ and these organizations emphasize the necessity of collecting appropriate reference data and ensuring the data apply to the actual source.^(8)^ In this context, important documents (e.g., TRS‐430,^(7)^ ESTRO Booklet No. 8,^(4)^ and the NCS Report No.13^(8)^) suggest minimum requirements for TPS commissioning, which are of great aid for medical physicists. Nevertheless, some of the tests mentioned in these publications may overlook some important ODIN in BTPSs. In addition, a recent publication^(9)^ suggested that the VID anisotropy table of an important TPS fabricant can produce ODIN regions up to 20% because of a lack of information in the angles of a high‐gradient zone, an issue that requires a more detailed review.

The aim of this work is to evaluate the performance of a commercial TPS with TG‐43 VID, analyze the possible ODINs and the implications in clinical standard treatments, and make a number of comments regarding certain commissioning tests widely recommended to detect these anomalies.

## II. MATERIALS AND METHODS

In the context of the acceptance and commissioning of a new BTPS in our institution, a BrachyVision 8.6 (Varian Medical Systems, Palo Alto, CA) was evaluated in the follow aspects:

### A. Reference data comparison

All input data were compared with the information published by Angelopoulos et al.^(10)^ and Sakellieu^(4)^ for a Varian 5 mm ^192^Ir HDR source (Vs2000), finding all possible areas with discrepancies over the 2% recommended by the AAPM.^(6)^

To compare different tables, it sometimes is necessary perform interpolations to match distinct axes. If another method is not indicated, we used a bilinear interpolation method. For example, to denote the array obtained from table alpha (depending on the c and d variables) and to make it compatible with the table beta (depending on the same factors), we use the notation $Z{\left(c,d\right)}_{\text{int}‐\text{alpha}2\text{beta}}$, which indicates “Z function table, interpolated from alpha to beta.”

### B. TPS evaluation in clinical conditions

Using the relevant clinical conditions, we found the percent dose‐rate differences between the dose rate calculated for 362 points (see below) by commercial software (${\left(\overset{\cdot }{D}(y,z)/S_{K}\right)}_{\text{TPS}}$) loaded with TG‐43 VID and the same points calculated with in‐house developed code (MATLAB 2009; MathWorks, Natick, MA) loaded with full TG‐43 data found in used references (${\left(\overset{\cdot }{D}(y,\;z)/S_{K}\right)}_{\text{code}}$). To ensure the reliability of our code, we compared ${\left(\overset{\cdot }{D}(y,\;z)/S_{K}\right)}_{\text{code}}$ with respect to the ESTRO along‐away dose rate table (${\left(\overset{\cdot }{D}(y,z)/S_{K}\right)}_{\text{ESTRO}}$) for the Vs2000 source, which consists of a point array in y (axial source axis) and z (long source axis) coordinates with 362 reference points centered in the middle of the active core. Moreover, to analyze the plausibility of the reference data election, we performed a short comparison with respect to other references regarding the source under study.^11^, ^12^, ^13^

**Figure 1:**
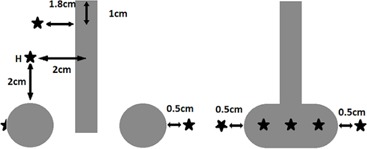
Image outlining the ABS points in a typical Fletcher treatment. On the left, a coronal plane with the points A (is the point H in the ABS nomenclature), tandem tip (1,8 cm), surface ovoid, and ovoid + 0.5 cm. On the right, a sagittal plane with rectal, bladder, and surface ovoid projection points.

The setups for clinical conditions are divided into the following:

- The one dwell position setup was designed to evaluate the TPS in the simplest configuration. This setup could simulate contact treatments of small skin lesions or partial breast irradiation with MammoSite.^(14)^
- The short applicator setup was designed to evaluate the TPS outcomes with one 3.5 cm‐long applicator (6 dwell positions). This setup can simulate a skin contact treatment and gynecological treatment as in the operated endometrial cancer or interstitial breast treatments with MammoSite.^(14)^
- Long applicator setup: This test was designed to evaluate the TPS with one 9‐cm long applicator, simulating contact and cervix endocavitary treatments, among others.
- Fletcher setup: Simulating typical Fletcher applicators (central tandems with two lateral ovoids), the tandem was 6 cm long, and the ovoid was calculated with a 2 cm diameter. In this test, additional points were added to the already existing 362 points to take into account the optimization points recommended by the American Brachytherapy Society (ABS),^(15)^ as we can see in Fig. 1. In addition to the coronal plane analysis, we present a tandem and ovoid‐centered sagittal plane to evaluate the dose to the organs at risk (rectum and bladder). For the analysis at the angles θ=0 and 180°, we used the anisotropy table published by Taylor and Rogers^(11)^ ($F{\left(r,\theta \right)}_{\text{Taylor}}$).
- Multiapplicators: This setup simulated the conditions of multiple‐applicator treatment as used in some interstitial techniques (e.g., prostate, breast, and cervix). As in the Fletcher setup, we performed the evaluation using the most relevant coronal and sagittal planes.

For simplicity of computations, all dwell steps are in the plane or in an orthogonal disposition with respect to ${\left(\overset{\cdot }{D}(y,z)/S_{K}\right)}_{\text{ESTRO}}$. In addition, to calculate the dose rate in a point with polar coordinates r and θ (r is the distance between the center of the source and the point and θ is the angle with respect to the long axis of the source), the TPS and the in‐house code use the TG‐43 line source approximation (LSA) formalism expressed as

(1) $$\dot{D}(r,\theta )=S_{k}\Lambda \frac{G_{L}(r,\theta )}{G_{L}(r_{0},\theta_{0})}g_{L}(r)F(r,\theta )$$

where $S_{K}$ is the air kerma strength of the source, *Λ* is the dose rate constant, $g_{L}(r)$ is the radial dose function for a LSA, F(r,θ) is the anisotropy function, and $G_{L}(r,\theta )$ is the LSA geometry function that takes into account the variation of the relative dose due only to the spatial distribution of the radioactive material within the source that in our code is calculated according to the equation proposed by TG‐43U1^(6)^ and by the AAPM‐ESTRO report.^(16)^

(2) $$\begin{array}{l} G_{L}(r,\theta )=\\ \{\begin{array}{ll} \frac{{\text{cos}}^{-1}\left(\frac{r\;\text{cos}\theta -\frac{L}{2}}{\sqrt{r^{2}+{\left(\frac{L}{2}\right)}^{2}-Lr\text{cos}\theta }}\right)-{\text{cos}}^{-1}\left(\frac{r\;\text{cos}\theta +\frac{L}{2}}{\sqrt{r^{2}+{\left(\frac{L}{2}\right)}^{2}-Lr\text{cos}\theta }}\right)}{Lr\text{sin}\theta }, & if\;\theta \neq 0\\ {\left(r^{2}-\frac{L^{2}}{4}\right)}^{-1}, & if\;\theta =0 \end{array} \end{array}$$

To obtain the radial dose function, we used a Meissberger fourth‐order polynomial fit supplied in the TPS.

We use the gamma analysis^(17)^ to evaluate the clinical cases with major discrepancies. For this purpose, two brachytherapy plans were made in the TPS (with a calculus grid size of 0.5 mm) for each selected case, with a $F{\left(r,\theta \right)}_{\text{VID}}$ and $F{\left(r,\theta \right)}_{\text{Taylor}}$, respectively. The dose planes exported from the TPS were analyzed by a commercial software (VeriSoft 4.2; PTW, Freiburg, Germany) using a distance to agreement (DTA) of 3, 2, and 1 mm and a d%L (local percentage dose difference) of 3%, 2%, and 1% in different combinations to evaluate the importance of such parameters in the analysis, without any lower dose analysis cutoff nor increased dose tolerance in lower dose regions.

## III. RESULTS

### A. Reference data comparison

**Figure 2:**
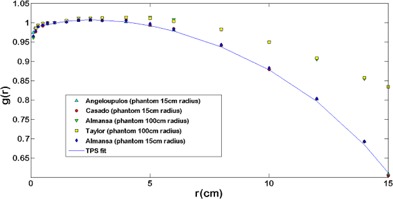
Comparisons among different Monte Carlo simulations of the g(r) function. It possible to observe a good agreement between simulations performed on similar phantom dimensions and the TPS fit. Comparing simulations with different phantom sizes (15 and 100 cm radius), an agreement better than 1.2% up to r=4 cm is observed. After that, the agreement becomes worse up 39% at r=15 cm.

BrachyVision uses TG‐43 LSA calculations for a 0.5 cm active length source. The dose rate constant Λ is 1.10 cGy/U, whereas the Λ value reported by Angelopoulos is 1.101 cGy/U. The g(r) polynomial fit supplied in the TPS agrees within 1.5% with the values provided by Angelopoulos et al.^(10)^ (Fig. 2).

**Figure 3:**
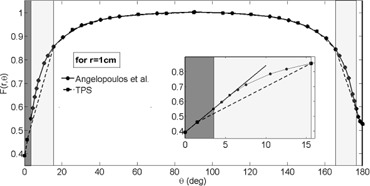
Comparative graph for F(1 cm,θ) between the TPS VID and Angelopoulos et al.^(10)^ publication data. In the dark zone, we can see the zone with a more than 2% difference. The darker zone highlights the extrapolated data. The box shows an amplified zone in which the lineal fit agreement with the VID extrapolated data can be observed.

With regard to the anisotropy function, the TPS was loaded with a VID table ($F{\left(r,\theta \right)}_{\text{VID}}$) with 17 angles and 10 radial distances. In contrast, the reference data had 31 angles and 9 radial distances. It is a mandatory requirement of the software to enter values for the 0° and 180° (or 90° for sources with axial symmetry),^(18)^ but these values were not supplied by the reference data. Table 1 shows $F{\left(r,\theta \right)}_{\text{VID}}$ for $\theta =\;0^{{}^{\circ}}$, 1.5°, and 180° for different radii compared with a first‐order extrapolations approach using a lineal fit that takes into account the last two neighbors of the extremes angles tabulated using $F{\left(r,\theta \right)}_{\text{ref}}$ (Fig. 3). The values obtained were the same as that in the TPS for six points, whereas for eight points, the differences were better than 1.1%.

**Figure 4:**
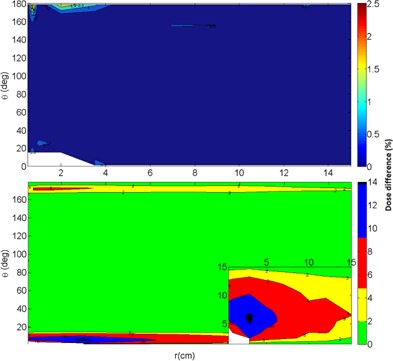
Absolute percent difference (top) between the $F{\left(r,\theta \right)}_{\text{VID}}$ and $F{\left(r,\theta \right)}_{\text{int}‐\text{ref}2\text{VID}}$. There is good agreement among both tables. Absolute percent difference (bottom) between the $F{\left(r,\theta \right)}_{\text{ref}}$ and $F{\left(r,\theta \right)}_{\text{int}‐\text{VID}2\text{ref}}$. There is a difference up to 14%.

Moreover, the $F{\left(r,\theta \right)}_{\text{VID}}$ has some radial distances (0.7, 3.5, and 13 cm), and the major portion of its angles (25.5°, 35.5°, 45.5°, 60.5°, 75.5°, 90°, 105.5°, 120.5°, 135.5°, 145.5°, and 155.5°) are not tabulated in the $F{\left(r,\theta \right)}_{\text{ref}}$. Figure 4 shows the absolute percent difference between $F{\left(r,\theta \right)}_{\text{int}‐\text{ref}2\text{VID}}$ and $F{\left(r,\theta \right)}_{\text{VID}}$, as observed, there agreement better than 0.5% ($\bar{x}=-0.06\%$, σ=0.38%) in a major portion of the matrix. Performing the inverse procedure (i.e., comparing the $F{\left(r,\theta \right)}_{\text{ref}}$ with $F{\left(r,\theta \right)}_{\text{int}‐\text{VID}2\text{ref}}$) we obtain approximately 14% for r=3 cm and $\theta =6.5^{{}^{\circ}}$ ($\bar{x}=-2.4\%$, σ=3.5%), as can see in Fig. 4.

**Table 1:** Comparison of the lineal extrapolated (LE) values for the angles not reported in the reference data

θ(deg)/r(cm)		*0.5*	*1.0*	*5.0*	*7.0*	*10*	*15*
0	TPS	0.434	0.392	0.443	0.512	0.588	0.673
	LE	0.434	0.392	0.444	0.512	0.588	0.674
1.5	TPS	0.498	0.460	${-}^{a}$	${-}^{a}$	${-}^{a}$	${-}^{a}$
	LE	0.498	0.459	${-}^{a}$	${-}^{a}$	${-}^{a}$	${-}^{a}$
180	TPS	0.559	0.526	0.603	0.652	0.716	0.714
	LE	0.553	0.526	0.609	0.648	0.717	0.717

a ^a^ Value is in the reference data and the same as in the TPS VID.

### B. TPS evaluation in clinical conditions

#### B.1 In‐house code evaluation

**Figure 5:**
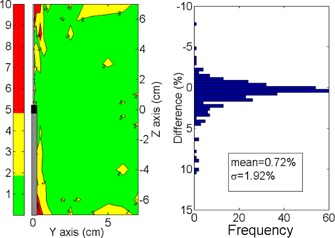
Absolute percent dose differences distribution between ${\left(\overset{\cdot }{D}(y,\;z)/S_{K}\right)}_{\text{code}}$ and ${\left(\overset{\cdot }{D}(y,z)/S_{K}\right)}_{\text{ESTRO}}$. On the left is the spatial distribution. On the right is the frequency distribution.

Figure 5 shows the absolute percent difference distribution between ${\left(\overset{\cdot }{D}(y,\;z)/S_{K}\right)}_{\text{code}}$ and ${\left(\overset{\cdot }{D}(y,z)/S_{K}\right)}_{\text{ESTRO}}$, with a maximum discrepancy of approximately 10%. The frequency distribution of such differences, with a mean of 0.72% and a standard deviation (SD) of 1.92%, is also shown.

To determine the source of the discrepancies, an anisotropy table, $F{\left(y,z\right)}_{\text{ESTRO}}$, was obtained from the ESTRO dose rate table through the formal TG‐43 F(r,θ) definition:

(3) $$F{\left(r,\theta \right)}_{ESTRO}=\frac{{\left(\dot{D}(r,\theta )/S_{k}\right)}_{ESTRO}G_{L}(r,\theta_{0})}{{\left(\dot{D}(r,\theta_{0})/S_{k}\right)}_{ESTRO}G_{L}(r,\theta )}$$

To avoid interpolations in the finding of ${\left(\dot{D}(r,\theta_{0})/S_{k}\right)}_{\text{ESTRO}}$, we used the equation

(4) $$\frac{\dot{D}(r,\theta_{0})}{S_{k}}=\Lambda \frac{G_{L}(r,\theta_{0})}{G_{L}(r_{0},\theta_{0})}g_{L}(r)$$

which indicated an agreement with ${\left(\dot{D}(r,\theta_{0})/S_{k}\right)}_{\text{ESTRO}}$ better than 1.8%. Once $F{\left(y,z\right)}_{\text{ESTRO}}$ was determined (through Eq. (3)), it was possible to compare it with $F{\left(y,\;z\right)}_{\text{int}‐\text{ref}2\text{ESTRO}}$ (note the change in the coordinates), which indicated the exact same error distribution as in Fig. 5.

#### B.2 Clinical conditions

**Figure 6:**
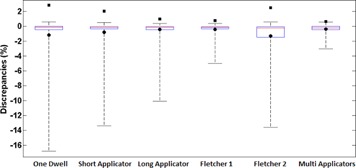
Amplitude (whiskers), 25th and 75th percentiles (lower and upper box limits), median (red line), mean (circles), and SDs (squares) of the discrepancies between ${\left(\overset{\cdot }{D}(y,\;z)/S_{K}\right)}_{\text{code}}$ and ${\left(\overset{\cdot }{D}(y,z)/S_{K}\right)}_{\text{TPS}}$ for each clinical setup evaluated.

Figure 6 shows the amplitude (whiskers), median (line on the box), 25th and 75th percentiles (lower and upper limits of boxes, respectively), mean (circles), and SD (squares) of the differences between ${\left(\overset{\cdot }{D}(y,z)/S_{K}\right)}_{\text{TPS}}$ and ${\left(\overset{\cdot }{D}(y,\;z)/S_{K}\right)}_{\text{code}}$ for the standard clinical setups. 7, 8, 9 show the most representative spatial distribution of the discrepancies around the applicators. Two ODIN regions can clearly been observed: one near the tip of the source and other on the vector cable. All ODIN points within 15° of the source long axis, which encompasses approximately 17% of the surrounding volume. The maximum discrepancy was found for the one dwell step configuration (−16.8%) at the point (0.25 cm, −2cm), which corresponds to the polar coordinates (2 cm,7°). The lowest differences was found for the multi‐applicator setup.

**Figure 7:**
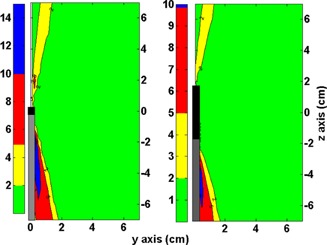
On the left side is the absolute percent dose difference between ${\left(\overset{\cdot }{D}(y,z)/S_{K}\right)}_{\text{TPS}}$ and ${\left(\overset{\cdot }{D}(y,\;z)/S_{K}\right)}_{\text{code}}$ for the one dwell position setup. On the right side is the absolute percent difference between ${\left(\overset{\cdot }{D}(y,z)/S_{K}\right)}_{\text{TPS}}$ and ${\left(\overset{\cdot }{D}(y,\;z)/S_{K}\right)}_{\text{code}}$ for the short applicator setup. Black and gray rectangles represent the source position and the vector cable, respectively.

**Figure 8:**
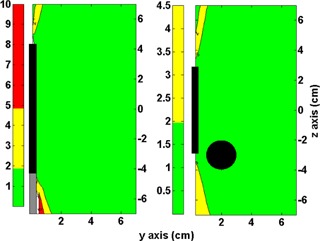
On the left side is the absolute percent dose difference between ${\left(\overset{\cdot }{D}(y,z)/S_{K}\right)}_{\text{TPS}}$ and ${\left(\overset{\cdot }{D}(y,\;z)/S_{K}\right)}_{\text{code}}$ for the large applicator setup. On the right side is the absolute percent difference between ${\left(\overset{\cdot }{D}(y,z)/S_{K}\right)}_{\text{TPS}}$ and ${\left(\overset{\cdot }{D}(y,\;z)/S_{K}\right)}_{\text{code}}$ for the Fletcher setup in a coronal analysis. Black and gray rectangles represent the applicators and the vector cable position, respectively.

Table 2 shows the results of the gamma analysis for one dwell and Fletcher setup (Fig. 10). For the one dwell setup, the origin of the dose plane is in the center of the source (coronal plane). Passing rates of 99.8%, 99.1%, and 90.3% for 3%L/3 mm, 2%L/2 mm, and 1%L/1 mm, respectively, were observed. In the case of Fletcher setup, the origin of the dose plane was in the center of the ovoid (sagittal plane). Passing rates of 100% (35 failed points), 99.9%, and 97.5% for 3%L/3 mm, 2%L/2 mm, and 1%L/1 mm, respectively, were observed.

**Figure 9:**
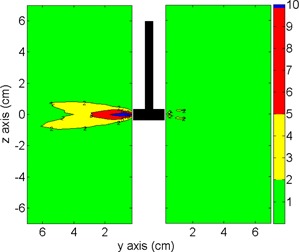
Fletcher setup analysis of the sagittal plane centered on the ovoid axis. On the left image is the absolute percent dose difference between ${\left(\overset{\cdot }{D}(y,z)/S_{K}\right)}_{\text{TPS}}$ and ${\left(\dot{D}(y,z)/S_{k}\right)}_{\text{code}}$ on the bladder side. On the right image is the absolute percent difference between ${\left(\overset{\cdot }{D}(y,z)/S_{K}\right)}_{\text{TPS}}$ and ${\left(\dot{D}(y,z)/S_{k}\right)}_{\text{code}}$ on the rectum side. Black rectangles represent the tandem and ovoid position.

**Table 2:** Results of the gamma analysis for one dwell setup (ODS) and Fletcher setup (FS)

*Evaluated Setup*	*Evaluated Points*	*Passed points (%)*	*Gamma Mean*	*Gamma Min*.	*Gamma Max*.	*Gamma Median*
ODS 1%L/1mm	251001	226675 (90.3)	0.515	0.0	16.626	0.150
ODS 1%L/2mm	251001	243142 (96.9)	0.263	0.0	12.736	0.150
ODS 1%L/3mm	251001	248880 (99.2)	0.219	0.0	12.736	0.150
ODS 2%L/1mm	251001	236766 (94.3)	0.290	0.0	8.313	0.075
ODS 2%L/2mm	251001	248619 (99.1)	0.169	0.0	6.368	0.075
ODS 2%L/3mm	251001	250339 (99.7)	0.141	0.0	6.368	0.075
ODS 3%L/1mm	251001	238881 (95.2)	0.213	0.0	5.542	0.050
ODS 3%L/2mm	251001	249586 (99.4)	0.133	0.0	4.245	0.050
ODS 3%L/3mm	251001	250581 (99.8)	0.111	0.0	4.245	0.050
FS 1%L/1mm	251001	244653 (97.5)	0.188	0.0	14.703	0.088
FS 1%L/2mm	251001	249490 (99.4)	0.152	0.0	10.677	0.088
FS 1%L/3mm	251001	250233 (99.7)	0.136	0.0	5.105	0.088
FS 2%L/1mm	251001	249513 (99.4)	0.115	0.0	7.352	0.044
FS 2%L/2mm	251001	250659 (99.9)	0.092	0.0	5.395	0.044
FS 2%L/3mm	251001	250843 (99.9)	0.082	0.0	2.680	0.044
FS 3%L/1mm	251001	250664 (99.9)	0.087	0.0	4.901	0.029
FS 3%L/2mm	251001	250898 (100)	0.070	0.0	3.659	0.029
FS 3%L/3mm	251001	250966 (100)	0.061	0.0	1.812	0.029

**Figure 10:**
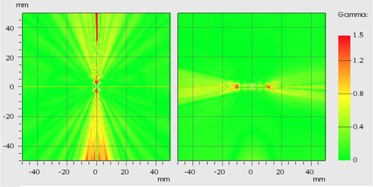
Gamma distribution (2%L/2 mm). On the left image, the coronal plane of one dwell setup. On the right, sagittal plane centered on one ovoid of the Fletcher setup.

## IV. DISCUSSION

### A. Reference data comparison

The finite capacities of informatics resources necessitate interpolations with the subsequent discrepancies between the TPS and proper published data. In this work, we use the ODIN concept to describe the dose discrepancy appearing when an interpolation has a disagreement over the 2% recommended by the AAPM.^(6)^ However, although these discrepancies may occur in the context of a physics‐controlled field, it is necessary to place these ODIN in the clinical context to evaluate their importance and achieve a major understanding of the global situation. The IAEA TRS‐430^(7)^ and the NCS Report No. 13^(8)^ recommend that the discrepancies between the TPS and the published data not exceed 5%. Accordingly, we can use these limits as the investigation and intervention levels, respectively.

ESTRO Booklet No. 8^(4)^ presents a full TG‐43 dosimetry set of the source under study, obtained from the Angelopoulos study^(10)^ and from L. Sakelliou (personal communications, 2003; a member of the same researcher group). Nevertheless, the dose rate table (along‐away) introduced has important differences (up to 10%) with respect to the Angelopoulos dosimetry set. This was concluded by comparing the $F{\left(y,\;z\right)}_{\text{int}‐\text{ref}2\text{estro}}$ and $F{\left(y,\;z\right)}_{\text{ESTRO}}$ through (3), (4). The comparison produces the same error distribution as seen in Fig. 5. It appears that such discrepancies come from some intrinsic difference between the Angelopoulos dosimetry set and the ${\left(\dot{D}(r,\theta_{0})/S_{k}\right)}_{\text{ESTRO}}$ data source; therefore, the use of this table requires careful revision. Given our findings above, we can conclude that our in‐house code works properly, despite the differences found (Fig. 5).

In the g(r) case, the TPS polynomial fit has good agreement with the Angelopoulos data, better than 1.5%. Though it is true that this is good agreement, it is necessary to consider that these parameters were obtained with a 30 cm diameter phantom. Figure 2 depicts the effect of the phantom size on the g(r), where agreement better than 2% is achieved only for r<4 cm among the simulations with different phantom sizes. Accordingly, it is imperative that this information be used in the dose evaluation for organs over 4 cm from the applicator.^13^, ^19^, ^20^

Concerning F(r,θ), the phantom size chosen has less influence in the simulation results than for g(r) because of its definition,^13^, ^19^ and it appears that the sphere is the best choice of phantom shape to calculate the F(r,θ).^(19)^ There was good agreement among different simulations, with discrepancies over 5% distributed in angles very close to 0° and 180° and for large r values (over 10 cm).^10^, ^11^, ^12^, ^13^

The differences shown in Fig. 7 are caused by a lack of entries for angles in high‐gradient zones in the $F{\left(r,\theta \right)}_{\text{VID}}$, as we can see in Fig. 3. The underestimation of the anisotropy factor by the lineal interpolation can increase up to 14%, and the differences over 2% cover the region between 1.5° to 15° near the vector cable and 167° to 176° near the source tip (for all r). From the 4, 7, there is a clear correlation between the lineal interpolation discrepancies and the dose differences in a one dwell position setup. Therefore, these dose discrepancy zones are ODIN regions.

### B. Clinical conditions

The results for the one dwell position setup has particular importance for MammoSite treatments using one dwell step,^14^, ^21^ where the ODIN regions may directly affect approximately 17% of the target volume. For a balloon of $70\;{\text{cm}}^{3}$ (approximately radius 2.6 cm approximately),^22^, ^23^ the target is exposed to the maximum discrepancies (over 10% of dose underestimation) shown in Fig. 7. In the case of contact treatments, it is quite common to use boluses or contact applicators between the source and the skin.^24^, ^25^, ^26^ Thus, it is possible that the ODIN regions avoid (with the source positioned parallel with respect to the skin surface) the target volume. Therefore, there are other factors with more influence with respect to the calculation dose, such as the lack of scatter effect, which may be responsible for dose overestimation of approximately 6% to 10%.^27^, ^28^, ^29^

In the short and large applicator setup, we can see how the addition of more dwell positions moves the ODIN regions to the peripheral zones (7, 8). The short applicator setup is usable in postoperative adjuvant therapy for endometrial cancer with a vaginal cylinder applicator,^(30)^ which generally has diameters from 1.5 to 4 cm and a dome‐shaped ending in the vaginal apex. In this setup, the major part of the ODIN region near the vector cable is inside of the cylinder and thus does not affect the target tissue. Nevertheless, the tip ODIN region compromises the dose calculation on the target volume (vaginal apex) and some organs at risk (sigmoid rectum).

In the gynecological treatments (e.g., cervix treatment with narrow vagina or with vaginal extension disease), using a large applicator setup with tandem and cylinder^(15)^ produces the same error distribution as the short applicators setup, with the cable ODIN region inside of the cylinder. When taking the GYN GEC ESTRO CTV volume delineation, only in case of voluminous initial disease with poor remission (at brachytherapy start) should the uterus be included in the intermediate risk CTV.^(31)^ In the authors' experience, even in that case, with an active length distribution of 4 cm in the vagina and 5 cm above the cervix, it is uncommon that the tip of the applicator is far enough inside of the CTV that tip ODIN will significantly affect the target dosimetry. As with the short applicator setup, the sigmoid rectum can be affected with a dose underestimation related to the tip ODIN zone.

The Fletcher setup in the coronal and sagittal plane (tandem centered) analysis indicated the same ODIN regions, all below the interventional 5% level. Nevertheless, on the tip, the ODIN zone may affect the organ at risk in the same manner as in the large applicator setup. In the case of the cable (tandem) ODIN region, it is inside of the vaginal cavity without affecting either the target volumes or the volumes of the organs at risk. When the sagittal plane is moved to the long ovoid axis, we obtain different distributions of the discrepancies (Fig. 9) depending on the side of the analysis. For the rectum side, a few points are over the 2%, which represents a good agreement between the TPS and our in‐house code. On the other hand, the bladder side displays important discrepancies, approximately up to 10% (at r=1.5 cm). As all ABS points are below the 0.5% difference level (including the rectum and bladder points), the results reported in this work demonstrate the manner in which such discrepancies can be hidden with 2D dosimetry, obviating distortions on the dose volume histogram (DVH) analysis, overall on the dose volume index as $D_{0.1\text{cc}}$, $D_{1\text{cc}}$, and $D_{2\text{cc}}$ or V75% or V70%.

The results indicate that the multi‐applicator setup produces differences below the interventional value of 5%(≤3%). Interpreting this information in a prostate implant, we can conclude that no relevant differences are found in the regions corresponding to the rectum and bladder, overall, when the volumes of the organs at risk are delineated 9 mm above and below the target volume.^(32)^ Nevertheless, a dose underestimation for the zone corresponding to the penile bulb between 2% and 3% may be observed.

Endovascular treatments are a special case. ESTRO Booklet No. 8^(4)^ suggests that the standard equipment mentioned in that publication, with the TG‐43 parameters provided, are suitable to perform treatment planning in all general endoluminal procedures. Nevertheless, as the dose prescription is made frequently in a millimeter range (often 2 or 3 mm),^24^, ^33^ the dose distribution close to the source and the geometric uncertainties present a great challenge for the medical physicist. Therefore, the physicist should increase efforts to reduce any discrepancies between the TPS and the reference source.^(34)^ For g(r), the major discrepancy between the Angelopoulos data and the polynomial fit were at 1 mm from the source. Here, it is recommend to use published data using log‐lineal interpolations.^(16)^ Pujades‐Claumarchirant et al.^(35)^ recommend the use of an anisotropy table with steps of 1° to 2° to obtain a discrepancy below the 0.5% (for a ${\;}^{192}\text{Ir}$ Flexisource); however, this recommendation may require revision for other ${\;}^{192}\text{Ir}$ sources.

The gamma analysis shows very good results with passing rate metrics commonly used in external beam radiation therapy. The TG‐186 of the AAPM^(36)^ recognize the necessity of define a dose accuracy tolerance requirements considering a gamma‐index metrics for brachytherapy as an important first step, proposing an incipient criterion of 2%/2 mm with a 99% pass rate for clinically relevant points. On the other hand, a growing amount of literature is warning us about how the gamma analysis may overlook important errors in the TPS causing nonnegligible deviations in the DVH.^37^, ^38^, ^39^, ^40^ It should be noted that the ODIN problems here described, correspond to a systematic error similar to those tested (real or induced for the purpose of sensitivity analysis) in some publications cited above. Therefore, we need to perform a very careful evaluation in the commissioning process, where the medical physicist must ensure that the good results obtained in the gamma analysis excluded the possibility of noticeable effects in the DVH (or some important indexes extracted from it (i.e., $D_{2\text{cc}}$) caused by systematic errors. As the AAPM propose, the gamma analysis is a potential area of research in brachytherapy, which needs further validation.^(36)^

Despite the dose differences found (over the international recommendations), the gamma analysis shows results in agreement with the criterion proposed by the TG‐186.^(36)^ Therefore, further studies are required to exclude the possibility of undesirable effects in the DVH caused by ODIN in the $F{\left(r,\theta \right)}_{\text{VID}}$, associating the conclusions obtained with the sensibility of the gamma analysis, emphasizing in the standard treatments most affected by this error (i.e., one dwell and Fletcher setup). In this sense, it is necessary to pay special attention with regard to the metric used since, according to the results shown in Table 2, the DTA would be the most important factor in the analysis due to the high dose gradient inherent to the brachytherapy sources.

### C. Comments on QA procedures

It is clear that the major contributions to the discrepancies in this case come from the VID anisotropy table, specifically due to the lack of information for angles $<15^{{}^{\circ}}$. All recommendations advise a careful revision and comparison of the VID to a proper reference publication as part of a good practice in the acceptance commissioning process.^3^, ^7^, ^8^ Nevertheless, this is a not straightforward procedure because initially a VID set could appear as reasonable sample of the full reference data. Pujades‐Claumarchirant and colleagues^(35)^ and the AAPM^(16)^ recommend angle samples from 1° to 2° to obtain a TPS performance below 2%, which is approximately the angle step in most published anisotropy tables. Lozano et al.^(41)^ examined the issue in a different way by finding the optimal radius and angles for a given number of entries using an optimization algorithm and achieving a difference below 2% for the same number of entries as the BrachyVision VID tables.

**Figure 11:**
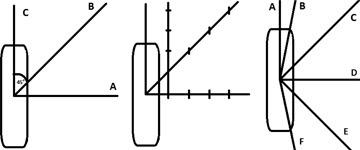
Tests recommended for the evaluation of the dose rate output from a BTPS. NCS report No. 13^(9)^ (left); ESTRO Booklet No. 8^(4)^ (middle); test recommended by this group with A at 0°, B at $0^{{}^{\circ}}<\text{ray}<20^{{}^{\circ}}$, C at 45°, D in 90°, E in 135°, and F between 160° and 180°.

NCS report No.13, ESTRO Booklet No. 8, and the TRS 430 recommend a point array to evaluate the TPS outcome (Fig. 11, left and middle). We can note this test is performed by assuming a symmetrical source with respect to it transverse axis; however, that condition is not a realistic assumption and, of course, is not supported by the Monte Carlo simulations.^10^, ^42^, ^43^ The last effect is produced by the cable perturbation of the water medium.^(9)^ Therefore, the F(r,θ) will have a steepest slope in the cable extreme. In that case, if the vendors use the same angular resolution for both sides, they could obtain different results in the interpolation compared with the reference data. In this situation, the Lozano approach^(42)^ could help the physicist in the choice of appropriate angles and radii to minimize the discrepancies.

With the understanding that the tests represented in Fig. 11 (left and middle) are the minimum recommended requirements and that the physicist should always perform more strict tests, if we compare the points array recommended with the results shown in Fig. 7, it is clear that this evaluation may be insufficient as a minimum requirement to aid in the finding of major disagreement. For that reason, we propose (assuming cylindrical symmetry) a half star pattern (Fig. 11 right) as a minimum requirement to evaluate the TPS dose rate calculation. Using the coordinates frame recommended by the AAPM^(16)^ (centered in the source active core with the 0° near to the source tip), we suggest the use of 6 rays placed at 0°, $0^{{}^{\circ}}<\text{ray}<15^{{}^{\circ}}$, 45°,90°, 135°, and $165^{{}^{\circ}}<\text{ray}<180^{{}^{\circ}}$. From our point of view, this is a stricter test that forces the TPS to display results in high‐gradient zones.

## V. CONCLUSIONS

The results of our evaluation are in agreement with those of other publications and suggest that the ODIN regions found in the BrachyVision loaded with $F{\left(r,\theta \right)}_{\text{VID}}$ has dosimetric implications in standard treatments, in certain cases affecting up 17% of the target volume (MammoSite case). With a maximum underestimation approximately 17%, the organs at risk could also be affected. From a QA point of view, we use values between 2% and 5% as reasonable tolerance levels. At these levels, medical physicists should investigate the source of such discrepancies and judge the clinical implication, adjusting the VID tables as necessary, using certain of the aforementioned methods. Although such dose differences are over the international recommendations, the gamma analysis shows results in agreement with insipient criterion; therefore, further studies are required to understand the clinical implications and exclude the possibility of find undesirable effects in the DVH in real treatments. We recommend the use of an half star pattern array of control points around the source to cover more complicated areas susceptible to being classified as ODIN regions.

## Supporting information

Supplementary Material
