# Supplementary material for: Brachytherapy treatment planning commissioning: effect of the election of proper bibliography and finite size of TG‐43 input data on standard treatments
Source: J Appl Clin Med Phys. 2015 Jul 8;16(4):3–17. doi: 10.1120/jacmp.v16i4.4730 (PMC5690009; doi:10.1120/jacmp.v16i4.4730)
Supplement: Supplementary file 1 — Supplementary Material [file ACM2-16-003-s001.doc]

1. Regard the correction made in the line 105, we want to cite the document 6 “Rivard M, Coursey B, DeWerd L, et al. Update of AAPM Task Group No. 43 Report: A revised AAPM protocol for brachytherapy dose calculations. Med Phys. 2004;31(3):633-674.” not the document 16 “Pérez-Calatayud J, Ballester F, Das R, et al. Dose calculation for photon-emitting brachytherapy sources with average energy higher than 50 keV: Report of the AAPM and ESTRO. Med Phys. 2012; 39(5):2904-2929.”
2. Regard the AAPM-ESTRO-ABG document (line 366), we avoid the problem change the sentence “proposed by the AAPM(36)” by “proposed in the TG-186(36)”
3. Regard the gamma analysis; we already include the additional 3/1, 3/2…2/3,2/1… etc. in the table 2, and a brief comment in the discussion (lines 368-370 of the corrected document).
4. All other changes were made according the recommendation.
